# Characterization of Zebrafish Models of Marinesco-Sjögren Syndrome

**DOI:** 10.1371/journal.pone.0165563

**Published:** 2016-10-28

**Authors:** Genri Kawahara, Yukiko K. Hayashi

**Affiliations:** Department of Pathophysiology, Tokyo Medical University, Tokyo, Japan; UNITED STATES

## Abstract

SIL1 is a nucleotide exchange factor for the endoplasmic reticulum chaperone, BiP. Mutations in the *SIL1* gene cause Marinesco-Sjögren syndrome (MSS), an autosomal recessive disease characterized by cerebellar ataxia, mental retardation, congenital cataracts, and myopathy. To create novel zebrafish models of MSS for therapeutic drug screening, we analyzed phenotypes in *sil1* knock down fish by two different antisense oligo morpholinos. Both *sil1* morphants had abnormal formation of muscle fibers and irregularity of the myosepta. Moreover, they showed smaller-sized eyes and loss of purkinje cells in cerebellar area compared to controls. Immunoblotting analysis revealed increased protein amounts of BiP, lipidated LC3, and caspase 3. These data supported that the *sil1* morphants can represent mimicking phenotypes of human MSS. The sil1 morphants phenocopy the human MSS disease pathology and are a good animal model for therapeutic studies.

## Introduction

Marinesco-Sjögren syndrome (MSS; OMIM 248800) is an autosomal recessive disorder clinically characterized by cerebellar ataxia, mental retardation, congenital cataracts, and progressive muscle weakness [1–4]. Mutations in *SIL1* (Gene ID: 64374) were reported to be causative for MSS [5, 6]. SIL1 is also known as binding immunoglobulin protein (BiP)-associated protein (BAP) [7]. BiP plays a key role in protein quality control as a heat shock protein (HSP) 70 chaperone family member located in the endoplasmic reticulum (ER). SIL1 regulates the ATPase cycle of BiP as an adenine nucleotide exchange factor [7, 8]. SIL1-null *woozy* mutant mice exhibit progressive ataxia caused by loss of Purkinje cells via ER stress, together with myopathy [9–12]. These reports suggest that SIL1 plays essential roles in ER function in multiple tissues.

Zebrafish is a useful model to elucidate the pathomechanisms of a lot of human diseases including muscular dystrophies [13–15]. The analyses of zebrafish models for human muscle disorders have been facilitated because of rapid development and easy-to-identify muscle structural abnormalities using birefringence assay [16]. They are easily analyzed by the use of morpholinos during early development. Zebrafish models of muscle disease have also been used to rapid therapeutic drug screening for small molecules [17].

To generate a model fish of MSS for studies on the functions of zebrafish sil1 and for therapeutic drug screenings, morpholino antisense oligos that targeted zebrafish *sil1* mRNA were designed and injected into zebrafish eggs. Our data indicates the reduction of *sil1* expression causes the abnormal formation of muscles, small sized eyes and reduction of purkinje cells associated with increased marker proteins for ER-stress, autophagy, and apoptosis.

## Materials and Methods

### Fish and fish culture

Zebrafish (the AB line) were cultured at 28.5°C according to standard procedures [18] and standard criteria [19]. Fertilized eggs were collected and used for injection. For anesthesia, euthanasia we used tricaine solution. Our IACUC, Tokyo Medical University animal facility, indicates that they approved this research (the approval number:S28029).

### Morpholino oligonucleotide injections

Two different anti-sense morpholino oligo-nucleotides (MO) targeted to disrupt splicing of *sil1* mRNA were designed by Gene Tools LLC. The morpholino sequences were *sil1* MO1: 5’- CAGCATGGGAATAAACTCACCTGGT -3’ and *sil1* MO2: 5’- GGTGACTGTGTAAACAGAACAAATC-3’. Morpholinos (3 and 6 ng) were injected into the yolk of one- to two-cell stage embryos. For the injection control, we also used a control morpholino (CMO; standard control oligo, Gene Tools): 5’-CCTCTTACCTCAGTTACAATTTAT-3’.

To confirm with the effect of morpholino injections, zebrafish total RNA was extracted from 4 days post fertilization (dpf) embryos using RNeasy micro kit (Qiagen) and was converted to cDNA (Superscript III, Life science). To detect misspliced products, PCR was performed with ExTaq DNA Polymerase (Takara Bio) at 95°C for 30 s, 55°C for 30 s, and 72°C for 30 s for 35 cycles) with the following primer sets: *sil1* for exon 2 and 3, forward 5’-GGCAAACAAGTGGAGAGTCAG-3’, reverse 5’- ATGGCTGTCCATGTTCATCA-3’ and beta-actin, forward: 5’-ATCAGCAT GGCTTCTGCTCT-3’, reverse: 5’-CACCCTGGCTTACATTTTCAA-3’.

### Detection of muscle phenotype of *sil1* morphants by birefringence

Birefringence assay was performed to detect abnormal skeletal muscle structure by placing anesthesized fish on a polarizing filter and subsequently covering them with a second polarizing filter [17]. The filters were placed on an underlit dissection scope and the top-polarizing filter twisted until only the light refracting through the striated muscle was visible. Since the degree of birefringence is affected by the horizontal orientation of the fish, the fish were oscillated back and forth to account for differences in positioning.

### Measurement of size of eyes

The diameter of the eye of 4 dpf embryos was measured under a dissection scope (Olympus, SZX10) with DP controller (Olympus) software and ImageJ (http://imagej.en.softonic.com/).

### Zebrafish SIL1 antibody

A rabbit polyclonal antibody against zebrafish sil1 was produced by synthesized peptides (C+EDLEVFRPTDKWQTLRPGQ; 63–81) as an antigen (Sigma Aldrich). The synthesized peptides were also used to absorb the antibodies to confirm the specificity of this antibody.

### Immunohistochemistry

Whole fish embryos were fixed in 4% paraformaldehyde overnight at 4°C and stored in 100% methanol at -20°C. After rehydration with a 50% methanol in PBS and blocking with 2% casein in PBS containing 0.05% Tween 20 (PBS-T) to reduce non-specific binding, embryos were incubated separately with either anti-beta dystroglycan (1:100, Novocastra), anti-parvalbumin (1:1000, Chemicon), or anti-myosin heavy chain (F59, 1:25, Santa Cruz Biotechnology) antibodies at 4°C overnight. After washing several times, the samples were incubated with secondary antibodies (1:500, anti-mouse AlexaFluor 488, 1:500, anti-rabbit AlexaFluor 568, Invitrogen) and DAPI (DAKO) solution for 30 min. The stained embryos were observed under confocal microscopies (Leica LCM710).

### Western blotting analysis

Proteins were extracted from 20 embryos of 4 dpf in RIPA buffer (Sigma) containing protease inhibitors and phosphatase inhibitors (Roche). A total of 30 micrograms of proteins were separated by electrophoresis on 5–20% gradient Tris—glycine gels (Wako Chemicals) and transferred onto a PVDF membrane (Bio-Rad). After blocking the membrane in PBS-T containing 2% casein, blotted proteins were incubated with each primary antibody of anti-zebrafish sil1 (1:200), anti-tubulin (1:500, Cell Signaling Bio), anti-BiP (1:250, abcam), anti-LC3 (1:250, Cell Signaling Bio), anti-activated caspase 3 (1:250, abcam), and anti-beta actin (1:500, Sigma) at 4°C overnight. After washing, the membranes were incubated with horseradish peroxidase (HRP) secondary antibody (anti-rabbit or mouse IgG, 1:15,000, abcam). Proteins were detected using a western blotting detection kit (Bio-Rad). Relative band intensities were quantified by densitometry using ImageJ, and fold differences determined between each proteins/beta-actin ratio.

### Cloning of zebrafish *sil1* cDNA and *in vitro* transcription of RNAs

Primers used to amplify the full-length zebrafish *sil1* cDNA coding sequence (XM009291221) were forward; 5’-TCTTTTTGCAGGATCATGTTGACAAGT CGTGTAATG-3’, reverse; 5’-CGAATCGATGGGATCTTACACCTGTCGCATT TTTAC-3’. PCR was performed with Prime STAR HS DNA Polymerase (Takara) at 98°C for 15 s, 55°C for 15 s, 72°C for 1 min for 35 cycles according to the manufacturer’s protocol. The PCR product of the zebrafish *sil1* cDNA was cloned into pCS2+ vector using In-Fusion^®^ HD Cloning Kit (Takara). All PCR products and cloned fragments were sequenced by using sequencing primers: pCS2+ forward primer: 5’-CGGAGCAAGCTTGATTTAG-3’, pCS2+ reverse primer: 5’-CCCCCTGAACCTGAAACATAA-3’, zebrafish *sil1* forward primer2: 5’-CTCTGGACATGCTTGTGGAA-3’ and zebrafish *sil1* forward primer3: 5’-GCAGGTGGGACTGGACATTA-3’. Sequencing results were analyzed using NCBI database and program on BLAST. Zebrafish *sil1* mRNA was synthesized from Asp718-digested pCS2+ plasmids using the sp6 mMessage mMachine kit (Ambion) and 50 pg of mRNA was co-injected into 1-cell-stage embryos with zebrafish *sil1* MO1 and 2. Following injection, embryos were cultured in aquatic system at 28.5°C.

## Results

### Zebrafish *sil1* morphant morphology

**Fig 1:**
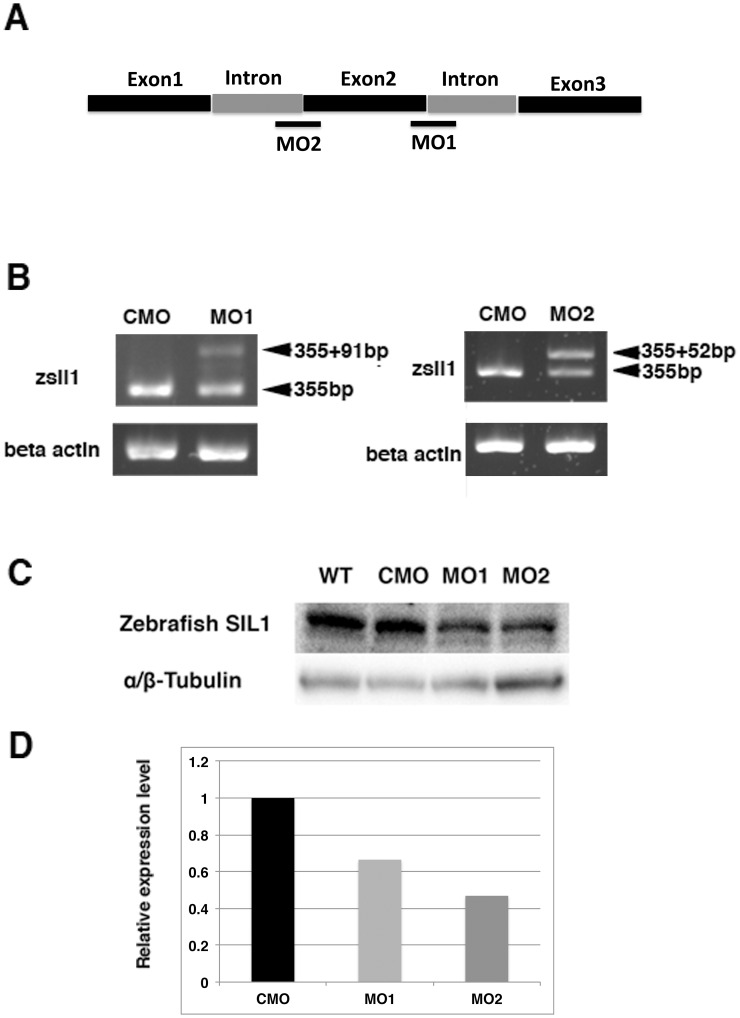
RT-PCR and protein expression of sil1 morpholino-injected embryos. A: Morpholino oligo-nucleotides target for zebrafish sil1. Two different anti-sense morpholino oligo-nucleotides targeted to disrupt splicing of *sil1* mRNA are designed by Gene Tools LLC. B: A single RT-PCR product of 355 bp is seen in 4 dpf embryos injected 3 ng of control morpholino (CMO), whereas 4 dpf embryos injected 3 ng of sil1 morpholino 1 (MO1) had two different sized products of 355 and 446 bp. Two RT-PCR products of 355 bp and 407 bp were detected in 4 dpf embryos injected morphant 2 (MO2). C: Western blotting analysis shows reduced amounts of sil1 protein extracted from 20 each of MO1- or MO2-injected morphants compared to 4 dpf wild-type embryos (WT) and CMO. D: Injections of MO1 or MO2 are effective to reduce the expression of sil1 protein to 66.5% (MO1, light gray) and 46.9% (MO2, gray) compared to CMO-injected fish (black), respectively.

Two different anti-sense morpholino oligonucleotides (MO) 1 and 2 were designed to disrupt splicing pattern of *sil1* mRNA during development (Fig 1A). The injected embryos were examined at 4 dpf. RT-PCR and sequence analysis confirmed that the MO1 and MO2 injection resulted in an in-frame insertion of a whole intron 2 (91 bps) and insertion of partial intron 2 (52 bps), respectively (Fig 1B).

To confirm the knock down effect to the expression of fish *sil1* by morpholino injections, endogenous zebrafish sil1 protein was analyzed with antibody against zebrafish sil1. In wild type and CMO injected fish, the sil1 antibody recognized a 52 kDa protein on western blots, a consistent size predicted by the fish sil1 sequence (Fig 1C). The expression of sil1 protein of MO1 or MO2 injected fishes was reduced to 66.5% and 46.9%, respectively compared to the wild type (Fig 1D). This result indicated that injections of MO1 or MO2 were effective to decrease the expression of sil1 protein.

### Birefringence assay

**Fig 2:**
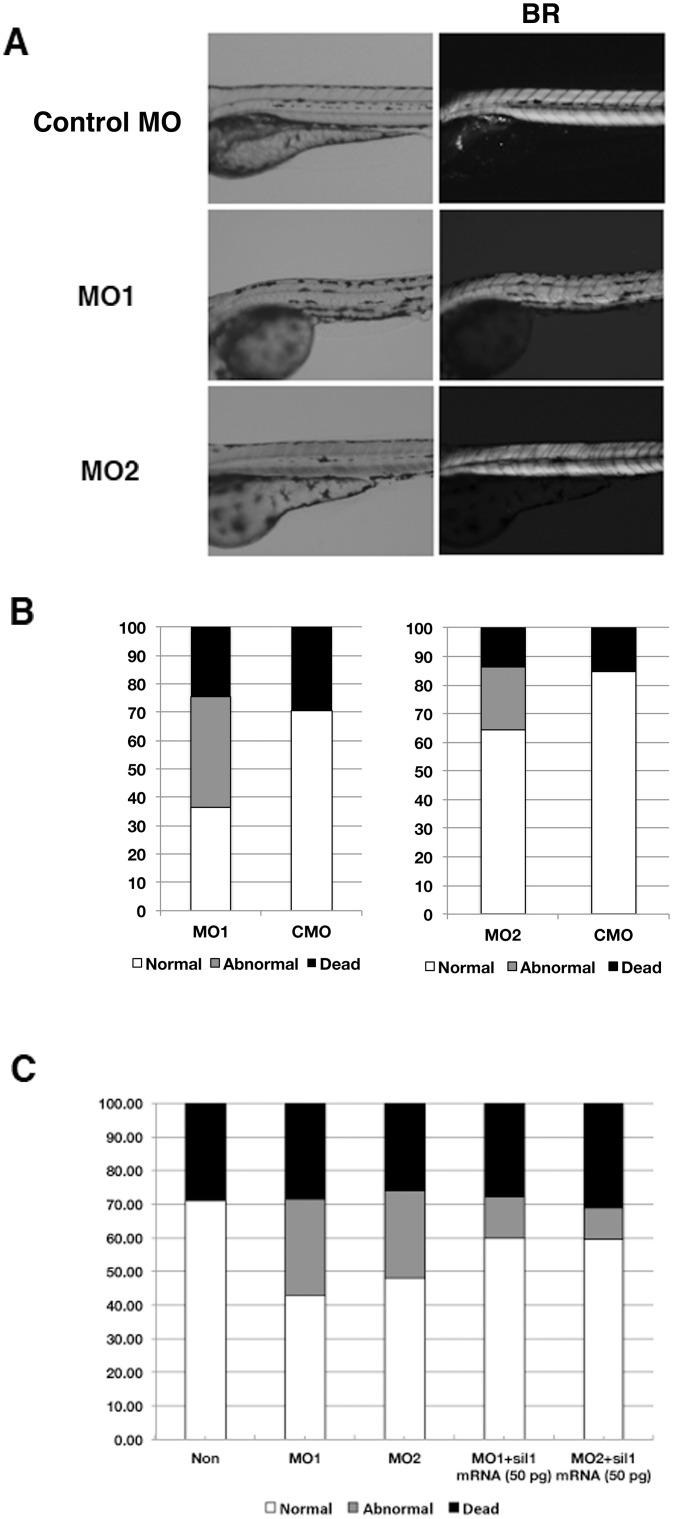
Altered skeletal muscle in sil1-morpholino injected fish. A: Left panels are pictures under bright field, and left panels are birefringence assay (BR). Abnormal structure of muscle is obvious in sil1 morpholino injected fish (MO1 and MO2) compared to control (CMO) under birefringence assay. B: Histogram of the percentage of normal, affected, and dead fish by morpholino-injection. Injection of 3 ng of MO1 or MO2 resulted in approximately 39.0% and 21.8% of injected embryos exhibiting reduced birefringence, respectively. C: Restoration of sil1 morphant with co-injection of fish sil1 mRNA (50 pg). Histogram of the percentage of dead, affected fish of morphants and recovered fish. Co-injection of zebrafish *sil1* mRNA with each MO (3 ng) rescued the phenotypes. White bar shows normal %, gray shows affected % and black shows dead fish %. Non: non injected fish, MO1: MO1 injected fish, MO2: MO2 injected fish.

Following injection of the two different morpholinos, some embryos showed abnormal shape visible upon light microscopy (Fig 2A). To better visualize the structure and organization of muscle fibers of the morphants, MO1, MO2 or CMO injected embryos and wild type embryos were analyzed by birefringence assays at 4 dpf (Fig 2A). The *sil1* morphant embryos were found to have markedly reduced normal patterns of birefringence compared to wild type and control morphants (Fig 2A). Injection of 3 ng of MO1 or MO2 resulted in approximately 39.0±1.8% and 21.8±3.1% of injected embryos exhibiting reduced birefringence, 36.5±4.7% and 64.5±1.6% were normal looking, and 24.5±4.7% and 13.7±1.6% of dead, respectively (Fig 2B). These percentages are an average of the results from three different experiments. The effects of morpholinos were dose-dependent and the ratio of abnormal embryos were increased when 6 ng of morpholinos were injected (S1 Fig). Importantly, co-injection of zebrafish *sil1* mRNA with each MOs rescued the phenotypes (Fig 2C).

### Immunohistochemistry of *sil1* morphants with muscle structure’s components

**Fig 3:**
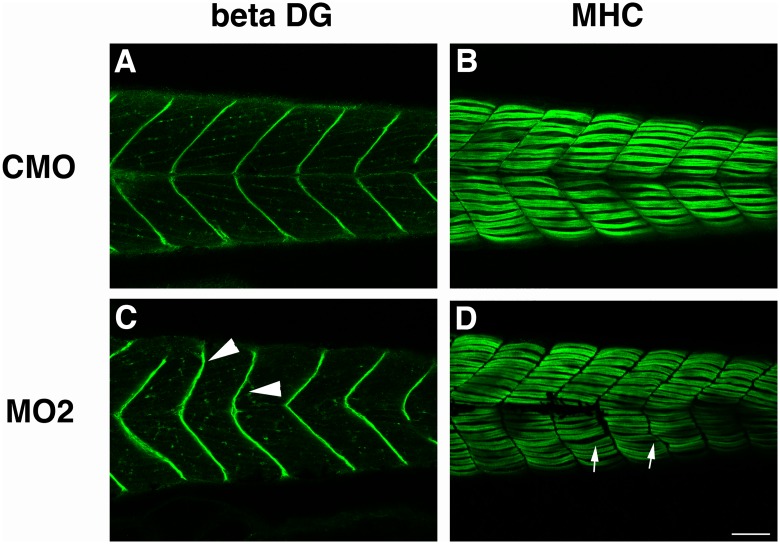
Immunohistochemistry of skeletal muscle tissue of morpholino-injected fish. Immunostaining of CMO injected fish (A, B) and sil1 morphant (MO2) injected fish (C, D) with antibodies against beta-dystroglycan (beta-DG) (A, C) and myosin heavy chain (MHC) (B, D). Beta-dystroglycan expression at the myosepta of MO2 injected 4 dpf embryos is misshapen and has a less clear v-shaped structure. Staining with anti-MHC indicated that formation of myofibers is disturbed in MO2. Arrowheads indicate the disturbance of myosepta in MO2-injected fish. Bar: 100 μm. Arrows indicate the abnormal structure of myofibers.

To examine the expression of muscle proteins, antibodies against beta-dystroglycan, laminin (data not shown), and myosin heavy chain (MHC, slow fibers) were used for immunohistochemistry. Beta-dystroglycan expression at the myosepta of MO1 or MO2 injected 4 dpf embryos was misshapen and had a less clear v-shaped structure as observed in wild type and CMO injected embryos (Fig 3A and 3C). Staining with anti-MHC indicated that formation of myofibers was disturbed in MO1 and 2 (in Fig 3B and 3D). This result is consistent with that observed by birefringence assay. Co-injection of zebrafish *sil1* mRNA with each MO reduced the number of fishes showing marked myofibril disruptions by anti-MHC stain.

### Abnormal size of eyes and reduction of purkinje cells in cerebellar area of *sil1* morphants

**Fig 4:**
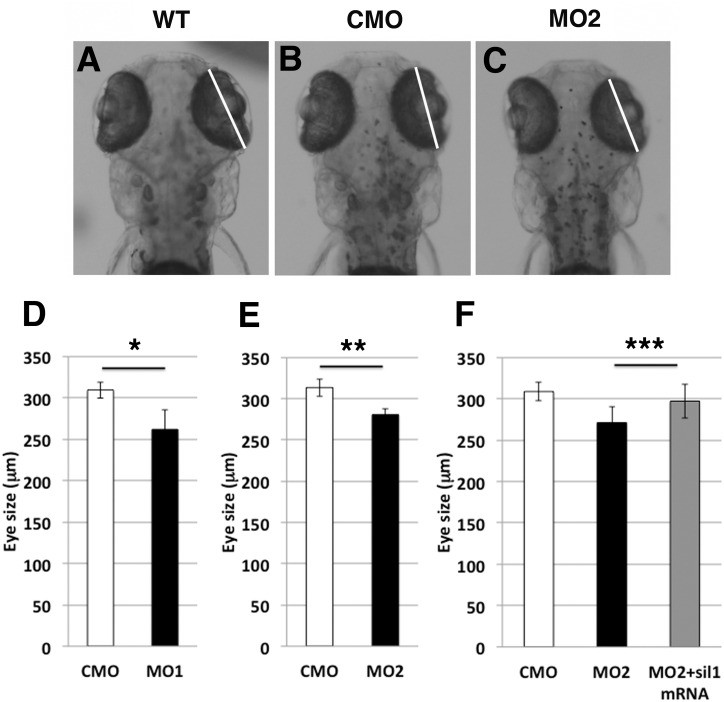
Smaller sized eyes in sil1-morpholino injected fish (4 dpf). The diameter of eyes in MO1 or MO2 injected 4 dpf embryos are smaller than those of CMO injected embryos. Co-injection of zebrafish *sil1* mRNA with each MOs rescues the eye size. Eye size is measured under a dissection scope with DP controller software and ImageJ. (A) wild type, (B) CMO injected fish, and (C) MO2 injected fish, showing white lines. Measurement of the diameter of eyes (D: MO1, E: MO2). (F): Restoration of eye diameter of sil1 morphant with co-injection of fish sil1 mRNA. Single asterisk indicates Student’s t-test p = 1.25E-07 (MO1; n = 19, CMO; n = 14), double asterisks indicate p = 7.36E-08 (MO2; n = 16, CMO; n = 28) and triple asterisks indicate p = 0.00441 (MO2; n = 19, MO2+sil1 mRNA 50 pg; n = 14, CMO; n = 14).

To examine influence of sil1 morpholinos to neural tissues, the size of eyes and number of purkinje cells in cerebellar area were analyzed. The diameter of eyes in MO1 or MO2 injected 4 dpf embryos was smaller than those of CMO injected embryos (Fig 4C, 4D and 4E). Co-injection of zebrafish *sil1* mRNA with each MOs rescued the eye size (Fig 4F).

**Fig 5:**
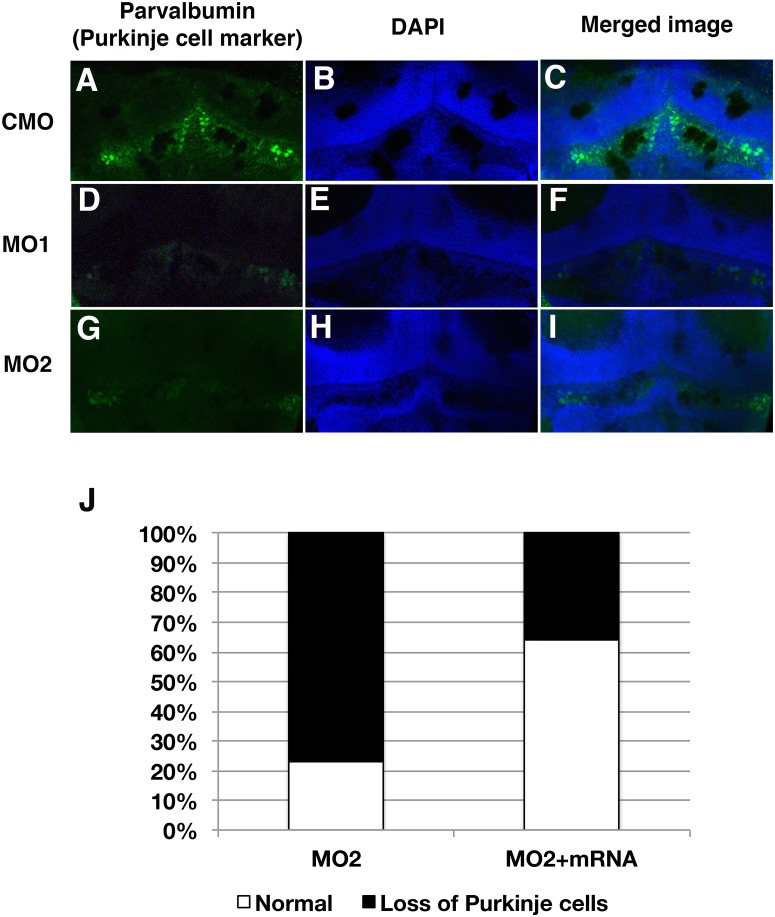
Staining of purkinje cells with anti-purvalbumin in cerebellar area. The number of purkinje cells detected with anti-parvalbumin shows reduced number of positive cells in MO1 or MO2 injected embryos. (A, B and C): CMO injected fish, (D, E and F): MO1 injected fish, (G, H and I): MO2 injected fish. (A, D and G: Staining Purkinje cell. (B, E and H): DAPI, nuclei. (C, F and I): Merged images. (J): Increased number of Purkinje cells in sil1 morphants with co-injection of fish sil1 mRNA (50 pg) from 23.1% (MO2, n = 13) to 64.3% (MO2+sil1 mRNA, n = 14).

The number of purkinje cells detected with anti-parvalbumin, a purkinje cell marker [20], showed reduced number of positive cells in MO1 or MO2 injected embryos compared to those of controls (Fig 5D and 5G). Co-injection of zebrafish *sil1* mRNA with each MO increased the number of positive cells in MO1 or MO2 injected embryos (MO2: 76.9%, n = 13, MO2+sil1 mRNA: 35.7%, n = 14) (Fig 5J).

### Increased expression of marker proteins of ER-stress, autophagy, and apoptosis

**Fig 6:**
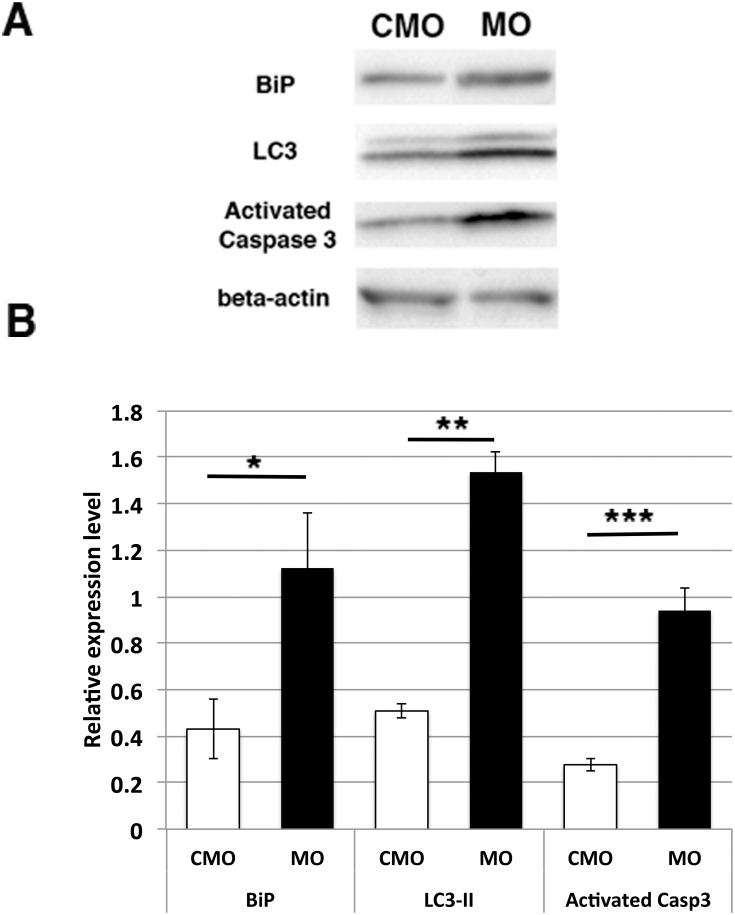
Protein expression analysis of sil1-morpholino injected fish. Western blot analysis of control morpholino (CMO) and sil1-morpholino2 injected fish (MO). The protein amounts of BiP, lipidated form of LC3 (LC3-II), and activated caspase 3 are significantly increased in *sil1* morphant embryos compared to those of CMO-injected embryos. A: Immunoblot with anti-BiP, LC3-II, activated-Caspase 3, and beta-actin. B: Relative expression level is analyzed via immunoblot (*p = 0.0226, ** p = 0.000102 and *** p = 0.000705, versus CMO, Student’s t-test, n = 3).

To examine expression levels of marker proteins of ER-stress, autophagy and apoptosis, western blotting was performed. The protein amounts of BiP, lipidated form of LC3 (LC3-II), and activated caspase 3 were significantly increased in *sil1* morphant embryos compared to those of CMO-injected embryos (Fig 6A and 6B).

## Discussion

Here, we showed a zebrafish is a good model of MSS, an autosomal recessive multisystem disorder characterized by cerebellar ataxia, mental retardation, cataract, and myopathy [1–6]. The sil1 morphants showed altered skeletal muscle structures, judged by unshapen myosepta and disturbed muscle fibers. In addition, the sil1 morphants have smaller sized eyes and loss of purkinje cells.

Our MSS zebrafish models display an increase in the levels of markers associated with ER stress, autophagy and apoptosis, which are similar to those observed in humans and a mouse model of MSS [9–11]. SIL1-deficiency is expected to reduce BiP activity, an ER chaperone protein, which leads to accumulation of abnormal proteins and subsequent ER stress, increased autophagy and apoptosis. In the reduced expression of sil1, morphants might have clear phenotypes in muscle, abnormal eye size and loss of purkinje cells via activated unfolded protein response, ER stress, autophagy and apoptosis. Interestingly, *sil1* morphants showed smaller-sized eyes instead of cataracts, which suggests that sil1 may have important roles in development of eyes in zebrafish. Recently SIL1 was reported to play a role in regulation of motor neuron subtype-selective ER stress in amyotrophic lateral sclerosis (ALS) [21]. Further analysis of the MSS model fish might reveal molecular crosstalk of ER stress, autophagy, and apoptosis in variable tissues and cells.

Further analysis of sil1 function in zebrafish should be undertaken CRISPR-Cas9 system for analysis of *sil1* functions and therapeutic intervention studies for MSS.

## Supporting Information

S1 Fig Histogram of the percentage of dead, affected fish of MO1 (A), MO2 (B) or control MO injected fish (3 ng and 6 ng). White bar shows normal %, gray shows affected % and black shows dead fish %. The effects of morpholinos are dose-dependent and the ratio of abnormal embryos are increased when 6 ng of morpholinos were injected.
(TIF)
