# Supplementary figures and images for: Characterization of Zebrafish Models of Marinesco-Sjögren Syndrome
Source: PLoS One. 2016 Oct 28;11(10):e0165563. doi: 10.1371/journal.pone.0165563 (PMC5085058; doi:10.1371/journal.pone.0165563)

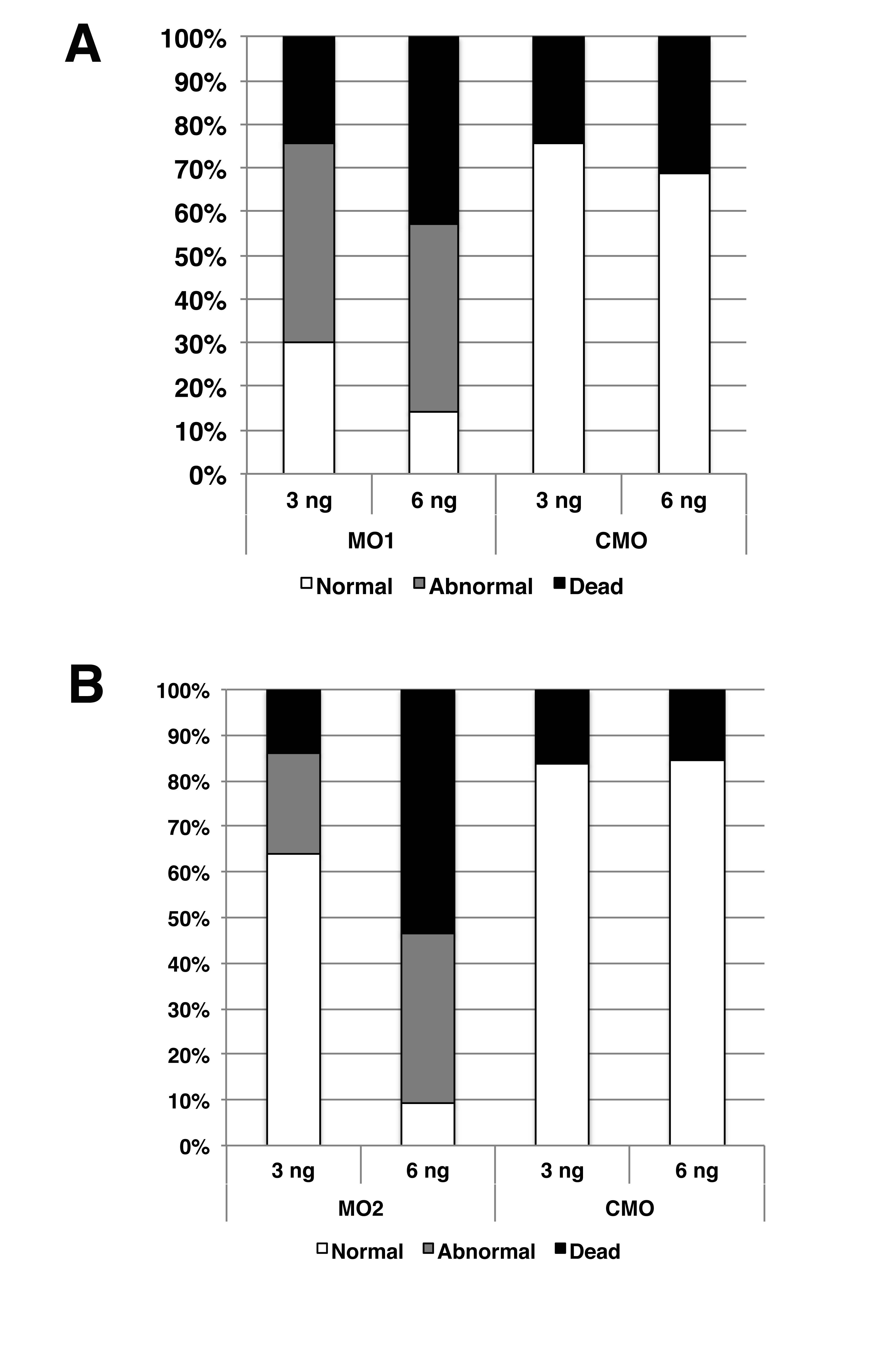

Supplement: S1 Fig — White bar shows normal %, gray shows affected % and black shows dead fish %. The effects of morpholinos are dose-dependent and the ratio of abnormal embryos are increased when 6 ng of morpholinos were injected. (TIF) [file pone.0165563.s001.tif]
